# Transitioning to a new Casemix grouper to fund long-term care homes in Ontario, Canada

**DOI:** 10.1186/1472-6963-11-S1-A25

**Published:** 2011-10-19

**Authors:** S Tsui, J Walker, P Temple, K Yu, C Hoy, M Alshurafa, J Walker

**Affiliations:** 1 Health Data Branch, Ministry of Health and Long-Term Care, Toronto, Ontario, L3M 1P8, Canada; 2 Dalla Lana School of Public Health, University of Toronto, Toronto, Ontario, M4S 2T9, Canada

## Introduction

Health system accountability and the capacity to care for the elderly with increasingly complex care needs is a challenge across many jurisdictions. The move to increasing accountability has necessitated greater and improved measurement of healthcare processes and outcomes. In 2005, the response by the Ontario Ministry of Health and Long-Term Care was to implement the Resident Assessment Instrument (RAI)-Minimum Data Set (MDS) 2.0. One purpose for the introduction of the assessment tool was to facilitate the transition to a new Casemix grouper with associated weights.

In long-term care in Ontario, homes are funded based on an envelope system. The majority of expenses related to resident care are provided for in the Nursing and Personal Care (NPC) envelope, which is 100% adjusted for resident acuity. The NPC envelope represents approximately 60% of envelope funding in the sector. Since 1993, this adjustment was based on the Alberta Resident Classification System (ARCS). Assessors from the Ministry measured ARCS once a year and, from these data, residents were classified into one of seven Casemix groups to formulate the province’s Casemix measurement. Long-term care (LTC) homes are funded based on their specific Casemix measure relative to the provincial Casemix measure.

Over time the validity of ARCS, and the ability to fairly and equitably distribute funding based on it, came into question. The introduction of the RAI-MDS 2.0, and the Casemix grouping algorithms associated with it, provided alternatives in order to improve the allocation of funding based on Casemix.

In collaboration with sector representatives, the Ministry determined that the Resource Utilization Groups (RUGs) 34-group model was best suited to Casemix-adjusted activity in long-term care. A transition model was developed and implemented in order to ensure system stability.

## Methods

The transition plan was developed in collaboration with the long-term care home sector. An advisory group, supported by a technical group, oversaw the development of options for the transition. The plan was based on the following principles:

1. Simplicity: the plan was to be as simple as possible in terms of the number and complexity of components. The fewer the components, and the less complex they were, the easier the plan would be to understand, communicate and implement.

2. Stability: the plan was to mitigate any instability that would be introduced into the system by switching to RUG III (e.g., corridors).

3. Transparency: the plan was to be transparent in that all components of the model were to be known and communicated to all stakeholders.

4. Sufficient notice: in advance of implementation of the transition plan, sufficient notice was to be provided to LTC homes and other stakeholders regarding implementation dates and impact of the plan on homes.

5. Revenue neutral to the province: Casemix transition was not to increase costs to the NPC envelope.

## Results

Due to the phased implementation of the MDS 2.0 assessment, the plan was implemented in two waves. In total, 217 homes were transitioned beginning April 2010 (Phase 1 – V homes). The remaining homes (400+) will transition starting April 2012.

The transition will last for three years for each wave. Therefore, the first group of homes will be through transition as of April 2013. During transition, a 5% corridor is being applied to the Casemix Index (CMI) so that the difference in CMI from year to year cannot be greater than, or less than, 5%. As a result of applying the corridor to the first group of homes, there were no homes whose funding decreased by more than 1% in the first year of transition.

Homes that began their transition in 2010 are now in their second year. The maximum decrease in funding after applying the corridor was greater than in the first year, although this was mitigated by an incremental funding allocation.

## Conclusions

To date, the plan has been successful in transitioning the long-term care sector to a new Casemix grouping methodology. Successful strategies employed included involving sector representatives in developing the plan; communicating widely; providing detailed face-to-face education on the plan, its components and impact; and keeping the plan simple.

This presentation will discuss the details of the development of the plan, as well as the year-two and -three estimates of funding changes as a result of the application of the corridor. Ongoing and emerging challenges will be described.

